# Altered B-Lymphocyte Homeostasis in Idiopathic Nephrotic Syndrome

**DOI:** 10.3389/fped.2019.00377

**Published:** 2019-10-09

**Authors:** Chen Ling, Xiaolin Wang, Zhi Chen, Jianfeng Fan, Qun Meng, Nan Zhou, Qiang Sun, Lin Hua, Jingang Gui, Xiaorong Liu

**Affiliations:** ^1^Beijing Key Laboratory of Pediatric Chronic Kidney Disease and Blood Purification, Department of Nephrology, National Center for Children's Health, Beijing Children's Hospital, Capital Medical University, Beijing, China; ^2^Key Laboratory of Major Diseases in Children, Ministry of Education, Beijing Children's Hospital, Capital Medical University, Beijing, China; ^3^Laboratory of Immunology, Beijing Pediatric Research Institute, Beijing Children's Hospital, Capital Medical University, Beijing, China; ^4^School of Biomedical Engineering, Capital Medical University, Beijing, China

**Keywords:** idiopathic nephrotic syndrome, B cells, children, transitional B cell, flow cytometry

## Abstract

**Background:** B-cell-deleted therapy has been successfully used for children with idiopathic nephrotic syndrome (INS), suggesting that B cells may be involved in the pathogenesis of INS. B cells are a heterogenous population comprised of subpopulations distinguished by their phenotypes. However, few studies have focused on the alteration of B-cell homeostasis in INS.

**Methods:** We measured the levels of B-cell subsets in the blood of 87 INS children via flow cytometry, prior to treatment with steroids. INS patients were divided into steroid-sensitive nephrotic syndrome (SSNS) and steroid-resistant nephrotic syndrome (SRNS) groups based on their sensitivities to steroids after a one-month follow-up. Subsequently, we compared these INS patients with age- and sex-matched patients with relapse (*n* = 35) and remissions (*n* = 32), as well as healthy controls (*n* = 75).

**Results:** We found that 65 SSNS patients exhibited an altered peripheral-blood B-cell-subset distribution, with increased levels of total, transitional, memory, IgM (immunoglobulin M) memory and switched-memory B cells compared to 22 SRNS patients. The proportion of total B cells was significantly higher in the SSNS group (22.1 ± 6.7% L, *p* < 0.001) than in the SRNS, remission, and control groups. In contrast, the levels of B-cell subsets in SRNS patients were generally the same as those in remission patients and healthy controls. Patients in relapse presented elevated memory B cells compared to those in other groups. The area under the ROC (receiver operating characteristic) curve of transition B cells at initial onset for the prediction of SSNS was 0.907 (95% confidence interval, 0.835–0.979). The analysis rendered an optimal cut-off value of 2.05 (% Lymphocyte) corresponding to 79.1% sensitivity and 90.9% specificity.

**Conclusions:** We observed and verified that B-cell subsets are significantly altered in children with SSNS. We propose that elevated transitional B cells may be a promising marker for predicting successful immunosuppressive therapy during the initial onset of INS. Further research is needed to determine the function of memory B cells in INS.

## Introduction

Idiopathic nephrotic syndrome (INS) is the most frequent glomerular disease during childhood. It is a clinical syndrome characterized by massive proteinuria, hypoalbuminemia, hyperlipidemia, and edema due to increased glomerular permeability. Although the role of B cells in several renal diseases is well-established (1), their contribution to the pathogenesis of INS is still being debated (2). The best supporting evidence for a role of B cells in INS comes from rituximab, a B-cell-specific antibody, which has been successfully used to treat patients with steroid-dependent or frequent relapse, and most of these patients have maintained remission in the absence of B cells (3–5). These findings imply that B cells are involved in the pathogenesis of INS.

B cells are a heterogeneous population that include different subsets characterized by their membrane phenotypes and/or cytokine-production profiles (6). B cells are capable of antibody production, antigen presentation, and immunomodulation. It has previously been reported—albeit debated—that CD19+ B cells increase during the acute phase of INS, but decrease to normal levels in the remission phase (7). It has also been reported that after treatment with rituximab, switched-memory B-cell recovery is associated with INS recurrence (8). In a recent study, elevated levels of memory B-cell subsets were considered to be closely related to the onset of INS (9). These studies suggest that different subpopulations of B cells play different roles in INS. However, few studies have focused on the alteration of B-cell homeostasis in children with INS.

Most INS is sensitive to prednisone treatment, and only a small number of patients exhibit steroid resistance (10). Steroid-resistant nephrotic syndrome (SRNS) is different from steroid-sensitive nephrotic syndrome (SSNS), and SRNS is currently recognized as a genetic disease or a disease due to other pathogenic factors. Therefore, we believe that SRNS and SSNS are also significantly different in terms of their corresponding subpopulations of B cells, and some B-cell subsets may serve as biomarkers for predicting one's sensitivity to steroid-therapy at the initial onset.

In the present study, we compared the distribution of B-cell subsets in the peripheral blood of SSNS and SRNS patients, as well as in healthy controls and in INS patients in relapse and remission. We hope that this investigation of altered B-cell subsets in INS may lead to the discovery of novel biomarkers for predicting INS-treatment responses.

## Materials and Methods

### Patients

This study was conducted between March 2018 and March 2019 at the National Center for Children's Health in China. Children with active nephrotic syndrome during the initial onset were recruited for the collection of blood samples before the treatment with prednisone. These children were followed up for at least 4 weeks. Patients who achieved remission with prednisone (60 mg/m^2^/day) within 4 weeks of treatment were classified as having SSNS, whereas those who did not respond were classified as having SRNS. Additionally, we included children with INS relapse and children with INS remission. Finally, 75 age- and sex-matched healthy children receiving routine medical examinations were included as healthy controls.

The diagnosis of INS was based on the presence of generalized edema, proteinuria (urine protein +++ or more by the heat-precipitation method and/or spot-urine protein/creatinine > 2 mg/mg), hypoalbuminemia (serum albumin < 2.5 g/dl) and hypercholesterolemia (serum cholesterol > 5.72 mmol/L). Patients were considered in relapse if they had edema, a serum albumin level of < 2.5 g/dl, and a urinary protein/creatinine (mg/mg) ratio of > 2.0, and they were considered to be in remission if they had no edema and their urinary protein/creatinine ratio was < 0.40 in a random urine sample.

Exclusion criteria were as follows: (1) age < 1 year old or > 10 years old; (2) positive family history of kidney disease; (3) systemic diseases, such as lupus and purpura; or (4) active infections, such as Epstein-Barr virus and cytomegalovirus.

Our treatment criterion followed the Improving Global Outcomes (KDIGO) guidelines of 2012 for kidney disease (11), which recommends the induction treatment of the initial episode of nephrotic syndrome in children with daily oral prednisone at 2 mg/kg/day (maximum 60 mg/day) for 4 weeks. No patients received or were ready to receive other forms of immunosuppression.

The study protocol was approved by the Capital Medical University Institutional Review Board, and informed consent was obtained from the parents or the authorized representative of each child.

### Cell Isolation

The blood of each child was collected in the morning after 1–2 days of hospitalization, and 3 mL of blood was collected in a heparin-anticoagulation tube. Blood samples were placed in a 4–6°C refrigerator and were analyzed within 6 h. Peripheral-blood mononuclear cells (PBMCs) were isolated from heparinized blood using the Ficoll-Hypaque (Axis-Shield, Oslo, Norway) density-gradient centrifugation method. Freshly isolated heparin-anticoagulated blood diluted in phosphate-buffered saline (PBS, 1:1) was layered on the surface of Ficoll at a ratio of 2:1 and was centrifuged at 1,600 g for 20 min at 20°C, with no breaks. The cells were harvested from the Ficoll interface.

### Flow Cytometry

To identify different B-cell subpopulations, PBMCs were stained with the following fluorochrome-conjugated antibodies: CD19-FITC (fluorescein isothiocyanate), CD24-PE (phycoerythrin), CD27-PE (phycoerythrin), CD38-PerCP (peridinin chlorophyll protein), IgM-APC (allophycocyanin), and IgD-PerCP (peridinin chlorophyll protein) (BD Biosciences, San Jose, CA, USA). The antibodies were then analyzed by a multicolor flow cytometer (FACSCalibur; BD Biosciences). Gated events (30,000) on living lymphocytes were analyzed for each sample. Four-color data acquisition was performed by FACS Calibur, and data were analyzed with CellQuest analysis software (BD Biosciences, San Diego, CA, USA).

**Figure 1:**
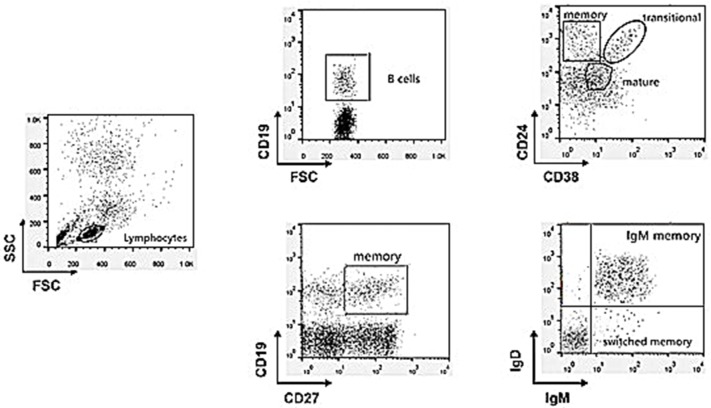
Gating strategy to discriminate the different B cell subpopulations by multicolor flow cytometry analysis.

Subsets of gated CD19^+^ (total) B cells were identified on the basis of the expression of surface markers as follows (Figure 1): transitional (CD24^high^CD38^high^), mature/naïve (CD24^low^CD38^intermediate^), and memory (CD24^high^ CD38^−^) or (CD27^+^) B cells. Memory B-cell subclasses (CD19^+^CD27^+^) were also defined as IgM memory (IgM^+^IgD^intermediate^) or switched memory (IgM^−^IgD^−^) and they were expressed as the percentage of total circulating lymphocytes (8).

### Statistical Analyses

Data were analyzed using SPSS v.19 (SPSS Inc., Chicago, IL, USA). Data are summarized as the mean ± SEM and median (interquartile range) for continuous variables, as well as the percentage and frequency for categorical variables. Statistically significant differences were analyzed using independent *t*-tests or Mann-Whitney *U* tests for continuous variables and chi-squared tests for categorical variables. A receiver operating characteristic (ROC) curve was constructed and the area under the ROC curve (AUC) was calculated to assess the predictive strength. Optimal cutoff points to maximize both sensitivity and specificity were also determined. Statistical tests were two-tailed, and a *P*-value of < 0.05 was considered statistically significant. Additionally, a *p*-value < 0.005 (i.e., 0.05/10) was used to indicate statistical significance after Bonferroni correction.

## Results

### Baseline Characteristics of the Study Population

The demographic characteristics of the subjects enrolled are presented in Table 1. A total of 156 patients with nephrotic syndrome were included in the study and were divided according to the stages of their diseases. Eighty-seven patients at disease onset (before any immunosuppressive treatment) were further divided according to their responses to prednisone (SSNS = 65, SRNS = 22). Additionally, there were 35 patients in relapse and 32 patients in stable (at least 1 month) remission. There was no difference in age or gender among the groups. Compared with that of the remission group and the control group, the albumin level in the disease-onset group and the relapse group was lower and the 24-h urine-protein level was higher. Serum-creatinine levels were normal for all patients and healthy controls.

**Table 1:** Characteristics of patients with nephrotic syndrome and controls.

**Parameter**	**Onset**		**Relapse (*n* = 35)**	**Remission (*n* = 3 2)**	**Health controls (*n* = 75)**
	**SS (*n* = 65)**	**SR (*n* = 22)**			
Age, years	5.2 ± 2.9	5.5 ± 4.3	5.6 ± 4.2	5.5 ± 2.8	5.2 ± 2.7
Sex, male	44 (67.7%)	16 (72.7%)	23 (65.7%)	22 (68.7%)	48 (64.0)%
Serum albumin, g/L	18.1 ± 5.8^‡‡^	20.9 ± 6.0^‡‡^	23.2 ± 8.3^‡‡^	35.4 ± 7.6^##^^,^ ^††^	–
Serum creatinine, g/L	32.8 ± 11.8	37.5 ± 15.7	37.2 ± 20.3	35.1 ± 7.0	–
24-h urine protein, mg/kg	132.1 ± 78.9^‡‡^	148.1 ± 103.5^‡‡^	135.3 ± 102.1^‡‡^	13.2 ± 9.5^##^^,^^††^	–
IgA, g/l	1.19 ± 0.78	1.10 ± 0.71	1.17 ± 0.62	1.31 ± 1.06	–
IgG, g/l	3.07 ± 2.9^#^^,^^†^^,^^‡‡^	3.98 ± 2.11^‡‡^	4.24 ± 2.41^‡‡^	6.98. ± 2.74^##^^,^^††^	–
IgM, g/l	1.57 ± 0.92	1.59 ± 0.94	1.50 ± 0.45	1.41 ± 0.57	–
IgE, g/l	216.2 (59.2, 537.8)^##^^,^^†^^,^^‡‡^	90.6 (42.4, 284.0)	45.0 (20.1, 346.5)	76.2 (55.7, 104.09)	–
T cell, %L	70.9 ± 8.9	71.6 ± 7.2	71.5 ± 8.2	71.4 ± 5.3	–
CD4+ T, %L	40.5 ± 8.2	36.9 ± 8.4	36.8 ± 8.2	39.1 ± 4.2	–
CD8+ T, %L	24.3 ± 6.0^#^^,^^†^^,^^‡^	29.9 ± 7.6	29.7 ± 7.0	27.9 ± 5.0	–
CD4/CD8,	1.8 ± 0.6^#^^,^^†^^,^^‡^	1.3 ± 0.5	1.2 ± 0.5	1.4 ± 0.3	–
Nature kill, %L	5.7 ± 3.0^##^^,^^††^^,^^‡‡^	8.0 ± 4.1^‡‡^^,^^†^	6.5 ± 3.8^‡‡^^,^^#^	13.5 ± 3.3	–
B cell, %L	22.1 ± 6.7^##^^,^^†^^,^^‡‡^^,^**	12.7 ± 6.1^†^	18.5 ± 7.4^#^^,^^‡‡^^,^*	13.7 ± 3.3^††^	14.1 ± 3.3^†^
Transitional B, %L	5.3 ± 3.8^##^^,^^††^^,^^‡‡^^,^**	2.0 ± 1.5^‡^	2.0 ± 1.8	2.5 ± 2.0^#^	2.0 ± 1.4
Mature B, %L	22.8 ± 9.6*	22.4 ± 8.9^‡^^,^*	23.7 ± 7.5^‡^^,^*	27.6 ± 8.0^#^^,^^†^	30.0 ± 11.0^#^^,^^†^
Memory B, %L	4.5 ± 2.4^††^^,^*	3.5 ± 2.0^††^^,^*	7.7 ± 5.5^##^^,^**^,^^‡‡^	4.6 ± 3.0^††^^,^*	2.8 ± 1.5^††^^,^^#^^,^^‡^
IgM memory B, %L	1.5 ± 0.8^††^^,^^‡^^,^*	1.0 ± 0.8^††^	1.9 ± 0.9^##^^,^^‡‡^^,^**	0.9 ± 0.5^††^	1.0 ± 0.5^††^
Switched memory B, %L	1.3 ± 0.8	1.0 ± 0.4^††^	1.4 ± 0.5^##^^,^^‡‡^^,^**	1.0 ± 0.3^††^	1.1 ± 0.4^††^

*SS, steroid sensitive; SR, steroid resistant; Ig, Immunoglobulin; %L, Percentage of lymphocytes*.

# p < 0.005;

## *p < 0.001, compared to SRNS patients at onset*.

† p < 0.005;

†† *p < 0.001, compared to patients in relapse*.

‡ p < 0.005;

‡‡ *p < 0.001, compared to patients in remission*.

* p < 0.005;

** *p < 0.001, compared to controls*.

In the relapse group and the remission group, all patients were treated with PDN. No children were treated with other immunosuppressants at the time of enrollment. For the IgA and IgM levels, there were no statistically significant differences among the groups. Additionally, the IgG level at the remission period was significantly lower than that during the active and relapse periods. The IgE level was significantly higher in SSNS patients than in other groups. The total T-cell ratio did not differ significantly among groups, and the CD4/CD8 ratio was slightly higher in the SSNS group than in the other populations. The proportion of NK cells was significantly lower in SSNS and relapsed groups than in other groups, and children in the SSNS group had a lower proportion of NK cells than those in the relapsed group.

### B-Cell Subsets Are Significantly Altered in Children With SSNS

**Figure 2:**
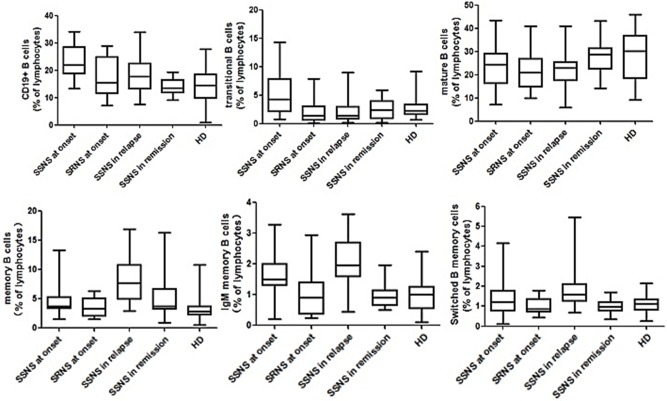
Multicolor flow cytometry analysis is circulating B cell subsets in NS patients and controls. B cells cell subsets from patients at disease onset (*n* = 87, in which SSNS = 65, SRNS = 22), in relapse (*n* = 35), in remission (*n* = 32), and healthy controls (*n* = 75) were compared with each other. All of them were expressed as percentages of total lymphocytes. Each box plot represents the median and the 25th and 75th centiles.

The proportion of total B cells was significantly higher in the SSNS group (22.1 ± 6.7% L, *p* < 0.001) than in the SRNS, remission, and control groups (Figure 2). Although prednisone was being applied, the children in the relapse group also had an increased proportion of total B cells compared with that in the SRNS, remission, and control groups (18.3 ± 5.5% L, *p* < 0.005). There were no statistically significant differences among the SRNS, remission and control groups in terms of the proportion of total B cells (*p* > 0.005).

The proportion of mature B cells did not differ significantly in SSNS, SRNS, and relapse groups (*p* > 0.005), but was slightly higher in the remission patients and healthy controls (*p* < 0.005).

The proportion of transition B cells was significantly higher in the SSNS group (5.3 ± 3.8% L, *p* < 0.001) than in the other groups, and was significantly lower in the relapse group and the remission group after treatment with PDN. These proportions were not different between the relapse, remission, and healthy control groups. There was no significant increase in the proportion of SRNS at the time of onset (*p* > 0.005).

Memory B cells did not increase significantly at the time of initial onset, but increased significantly in the relapse phase compared with those of other groups (7.7 ± 5.5% L, *p* < 0.001). This phenomenon was due to an increase in the proportion of IgM-memory B cells (1.93 ± 0.86% L) and switched-memory B cells (1.39 ± 0.51% L).

### Transition B Cells as a Biomarker for SSNS

**Figure 3:**
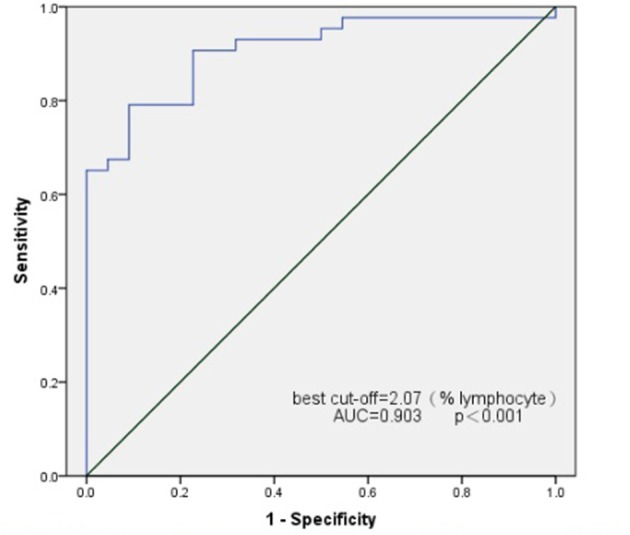
Receiver operating characteristics (ROC) curve analysis for transitional B cell as a marker for the identification of SSNS.

Transition B cells differed greatly in patients with newly diagnosed SSNS and SRNS. Hence, we attempted to use transition B cells as biomarkers to predict the outcome of PDN therapy. ROC curves were used to assess the potential utility of transition B-cell detection in patients with SSNS at the time of initial onset. The area under the ROC curve of transition B cells for the prediction of SSNS was 0.907 (95% confidence interval, 0.835–0.979). The analysis rendered an optimal cutoff value of 2.05% L corresponding to a 79.1% sensitivity and 90.9% specificity (Figure 3).

## Discussion

This cohort study from China confirmed that B-cell subsets are altered in an SSNS population in children. We tested 87 newly diagnosed INS children before any immunosuppressive treatment, and all patients were followed up for 1 month. Other groups were age- and sex-matched. According to the efficacy of prednisone, patients were divided into SSNS and SRNS groups.

An increased level of B cells has already been reported in SSNS, but conflicting results also exist (12). Colucci et al. suggested that prednisone treatment is the cause of these controversial conclusions (9). We confirmed the theory that CD19^+^ B cells were significantly elevated in initial-onset NS children before steroid treatment, which is consistent with reports by Colucci et al. (9). We also observed an increase in B cells in the relapse group compared with that in the remission group, but this increase was not as high as that of the SSNS group during first onset, which may be related to the effects of immunosuppressive therapy.

We further compared the changes in B-cell subsets in SSNS and SRNS groups during the first onset. We found that transitional B cells (CD19+CD24^hi^CD38^hi^) were significantly elevated in the SSNS population compared to those in the SRNS population. This is consistent with the results reported in a previous study (9). When using a cut-off value of 2.05 (% of lymphocytes), transitional B cells allowed observers to distinguish SSNS from SRNS with an AUC of 0.907, an optimized sensitivity of 79.1%, and a specificity of 90.9%. This suggests that transitional B cells may be a biomarker for early screening for steroid-sensitive nephrotic syndrome.

It is worth mentioning that in recent years, some transitional B cells have been found to secrete the anti-inflammatory factors, interleukin-10 (IL-10) and transforming growth factor-β (TGF-β), and to express inhibitory surface molecules, which are considered to have immunomodulatory effects (13). The importance of regulatory B cells has been shown in several autoimmune conditions. For example, a prolonged presence of transitional B cells has been associated with long-term non-relapsing of systemic lupus erythematosus (14). Moreover, transitional B lymphocytes are associated with protection from kidney-allograft rejection (15). The opposite result showed a positive correlation between CD38+CD19+ B cells and IL-10+CD19+ B cells and hepatitis B virus-associated membranous nephropathy (16). Our present study showed that transitional B cells exhibited a markedly abnormal increase in early disease, and that transitional B cells showed a markedly rapid and sustained decrease after steroid administration. Although our present study demonstrates that transitional B cells may be used as steroid-sensitive biomarkers in children with NS, it is necessary to further study the mechanisms of transition B-cell elevation and their role in the pathogenesis of SSNS.

Memory B cells are one of the focuses of current research on children with primary nephrotic syndrome. The first report by Colucci et al. showed that delayed reconstitution of memory B cells was protective against relapse after rituximab (8). This finding was partly confirmed by a study from India (17). However, Bhatia et al. observed that total B cells and memory B cells recovered earlier in relapsers, but Colucci et al. considered that this is related to the prolonged depletion of memory B cells rather than of total B cells. In our present study, memory B cells were significantly elevated in the relapse group and also in the SSNS group during onset. Overall, these phenomena may imply that memory B cells are involved in the pathogenesis of SSNS. One hypothesis of memory B cells in NS is that the Epstein-Barr Virus is able to establish latent benign infection in memory B cells that display phenotypes similar to those of antigen-selected memory B cells. A specific anti-EBNA1 antibody internalized in podocytes via the neonatal FC receptor might cross-react with a major podocyte protein (18). These possibilities have not been explored and should be investigated further.

The class conversion of immunoglobulins is the basis for the body to produce different classes of antibodies and serve different functions. We observed that SSNS had low IgG levels and high IgE levels. Following statistical analyses, we did not find any correlation between IgG and IgE with serum albumin and B-cell subsets. The role of antibodies in INS has been proposed by a number of clinical observations and experimental studies. Dantal et al. showed that a permeability factor inducing albuminuria may bind to an immunoglobulin and induce decreases of IgG (19). IgE is often associated with allergic diseases, and elevated IgE may be due to a response to elevated plasma IL-13 in allergic disease (20).

The current study also has limitations. First, although we showed that the percentage of transitional B cells appeared to be a marker of steroid sensitivity with reasonable sensitivity and specificity, this finding requires further verification in a separate group of recently diagnosed nephrotics. Second, although children in the relapsing and remission groups never received other immunosuppressive agents, their concurrent use of steroids may have had an effect on the levels of B cells and their subpopulations.

In conclusion, we validated that there is an alteration of B-cell subsets in children with SSNS, and that transition B cells may be a biomarker for SSNS during the initial onset before steroid treatment. Future studies should investigate the function of B-cell subsets at different periods of NS, which will help to further elucidate the pathogenesis of NS.

## Data Availability

All datasets generated for this study are included in the manuscript/supplementary files.

## Ethics Statement

The study protocol was approved by Beijing Children's Hospital at Capital Medical University. All procedures performed in our studies were in accordance with these ethical standards. All parents of the individual participants included in the study provided written informed consent.

## Author Contributions

CL and XL designed the research. CL wrote the paper. ZC, QS, JF, QM, and NZ were responsible for collecting clinical pathology. LH was responsible for statistical processing. XW and JG completed the laboratory work.

### Conflict of Interest Statement

The authors declare that the research was conducted in the absence of any commercial or financial relationships that could be construed as a potential conflict of interest.
